# Assessment of spatial learning and memory in the Barnes maze task in rodents—methodological consideration

**DOI:** 10.1007/s00210-018-1589-y

**Published:** 2018-11-23

**Authors:** Kinga Gawel, Ewa Gibula, Marta Marszalek-Grabska, Joanna Filarowska, Jolanta H. Kotlinska

**Affiliations:** 1 Department of Experimental and Clinical Pharmacology, Medical University, Jaczewskiego 8b, 20-090 Lublin, Poland; 2 Department of Pharmacology and Pharmacodynamics, Medical University, Chodzki 4a, 20-093 Lublin, Poland

**Keywords:** Barnes maze, Spatial memory, Apparatus, Procedure, Protocol, Rodents

## Abstract

Among the methods valuable for assessing spatial learning and memory impairments in rodents, the Barnes maze (BM) task deserves special attention. It is based on the assumption that the animal placed into the aversive environment should learn and remember the location of an escape box located below the surface of the platform. Different phases of the task allow to measure spatial learning, memory retrieval, and cognitive flexibility. Herein, we summarize current knowledge about the BM procedure, its variations and critical parameters measured in the task. We highlight confounding factors which should be taken into account when conducting BM task, discussing briefly its advantages and disadvantages. We then propose an extended version of the BM protocol which allows to measure different aspects of spatial learning and memory in rodents. We believe that this review will help to standardize the BM methodology across the laboratories and eventually make the results comparable.

## Introduction

There are several well-known animal tasks used for evaluating spatial learning and memory (Paul et al. 2009; Morellini 2013). These tests assume that the animal learns to solve a maze by using positive environmental (food, water, shelter) or negative environmental factors (immersion in water, intense light, noise, or air blast). In evaluating spatial learning and memory, researchers employ the radial arm maze test, spontaneous alternation and win-shift tests in the T and Y mazes, as well as spatial versions of the novel object recognition test, Morris water maze and Barnes maze (BM) test (Paul et al. 2009; Morellini 2013). The last test, described herein, is based on the assumption that the animal placed onto the surface of a platform should learn and remember the location of an escape box (i.e., safe shelter, dark and located mostly below the surface of the platform). The test consists of several phases (Paul et al. 2009). These include, firstly, a habituation phase (in which the animals are introduced to the environment), then an acquisition phase (during which the animals learn to find the location of the escape box). After a few trials, shorter latencies to reach an escape box are expected because in order to resolve the maze, the test animals change their applied strategy from random to spatial (see Table 1). Subsequently, the acquisition phase is followed by the acquisition probe trial which is carried out with a closed target hole and the time spent on the vicinity of the previously correct hole (or the correct zone) is measured. This allows an assessment of spatial memory retrieval (i.e., retention) (Paul et al. 2009). In turn, the second part of the test (reversal learning), by changing the shelter position, allows for assessing the cognitive flexibility in relearning a new location in a follow-up test (Paul et al. 2009; Stalnaker et al. 2009). The first part of the BM task, i.e., the acquisition phase followed by the acquisition probe trial, allows an evaluation of spatial learning and spatial memory. This part is believed to be associated with hippocampus function (Barnes 1979; Kennard and Woodruff-Pak 2011; Negrón-Oyarzo et al. 2015; Rodriguez et al. 2013), while the second part of the task (i.e., reversal learning trials) allows for the evaluation of the cognitive flexibility that is associated with frontal cortex function (Crews and Boettiger 2009; Chawla et al. 2017). Although BM is not as popular a task as the Morris water maze or radial arm maze, it possesses some advantages which make it a very attractive alternative, especially for the former. Moreover, the usefulness of this task is quite broad: from pharmacologically and genetically induced Alzheimer disease models, to other disease/injury models (e.g., after traumatic brain injury, Parkinson disease, lateral sclerosis) as well as to drugs (or drug regimens) which might improve or deteriorate the spatial learning and memory.

**Table 1:** The list of synonyms used in the current paper, as well as the summary of parameters and search strategies which can be measured in the BM task

Synonyms	✓ Escape box—safe shelter, goal box, target hole, tunnel
	✓ Habituation phase—shaping trial
	✓ Acquisition phase—learning phase, training phase
	✓ Probe trial—retention test, transfer test, retrieval
Escape latency	✓ Primary—time (s) needed to find and enter the escape tunnel—with head alone*^,#^
	✓ Total—time (s) needed to find and enter the escape tunnel—with whole body^#^
Error	✓ Reference—animal makes a nose and head deflection into a non-escape hole^#^
	✓ Working—animal makes a nose and head deflection into a non-escape hole already visited during the same trial^#^
	✓ Perseverative—search of the same hole without searching another hole in between
	✓ Semi-quantitative—for an animal who did not escape the maze within a given trial’s time limit
	✓ Primary—a nose and head deflection into a non-escape hole made by an animal before entering the escape tunnel with its head*^, #^
	✓ Total—a nose and head deflection into a non-escape hole made by an animal before entering the escape tunnel with whole body^#^
	✓ Hole deviation score—the number of holes between the first hole visited and the escape box
Path lengths	✓ Primary—animal path lengths (in cm) to reach a target hole with its head^#^
	✓ Total—animal path lengths (in cm) to reach a target hole with its whole body^#^
	✓ Peripheral—animal path lengths within 20 cm of the maze edge (in cm) (depending on the size of the apparatus)
	✓ Central—animal path lengths within a 40-cm radius from the center of the maze (in cm) (depending on the size of the apparatus)
Search strategy	✓ Direct (Spatial)—moving directly to the target hole or to 1–2 adjacent hole(s) before visiting the target^#^
	✓ Serial—the first visit to the target hole preceded by visiting at least two adjacent holes (but not adjacent to target hole) in serial manner, in clockwise or counter-clockwise direction^#^
	✓ Mixed (random)—hole searches separated by crossing through the center of the maze or unorganized search^#^
Running speed	The average speed (cm s^−1^) of an animal during the trial^#^

*The critical measures proposed by O’Leary and Brown (2013)

^#^The most frequently measured parameters in the literature

In this review, we describe the BM paradigm for measuring spatial learning and memory in rodents. We especially focus on methodological considerations when using this not new, but very interesting task. Thus, we aim to gather existing knowledge about the BM procedure. This was done because there are many approaches to conducting BM experiments, making comparisons of results quite challenging. Specifically, we summarize current knowledge about the maze test procedure, its modifications, variations and critical parameters measured in the task, as well as the confounding factors which should be taken into account when conducting the task. Moreover, both advantages and disadvantages are briefly discussed. There are some “gaps” in the literature with reference to the BM methodology. Thus, we highlight them to prompt researchers to fill these “gaps” in the future. Because the theoretical basis of BM neurobiology is beyond the scope of this review, we encourage the readers to familiarize themselves with other excellent reviews (Kapadia et al. 2016; Morellini 2013; Paul et al. 2009). Nevertheless, we believe that this review will help researchers (1) to choose a proper protocol (or its variations) which best fits the experimental aim, (2) to clarify all the confounding factors which may affect the performance of animals and subsequently interfere with the results, and (3) to standardize the methodology of BM across the laboratories, and therefore making the BM results comparable and reliable.

## Barnes maze

Although the BM task was first described by Carol Barnes in 1979, its usefulness was appreciated almost two decades later. There are few variations of the protocol described in the literature, but the basic protocol of the task allows to assess spatial working memory, spatial reference memory (short and/or long term) and cognitive flexibility. Thus, the flexibility of the BM test enables an assessment of diverse aspects of spatial learning and memory, using the same cohort of animals, in a relatively short period of time. Moreover, it is more easy to perform relative to other types of mazes, e.g., Morris water maze or radial arm maze (for the comparison of these three tasks, see Table 2, as well as Paul et al., 2009). Although some appetitive (after water or food deprivation) versions of the task have been described (Williams et al. 2003; Youn et al. 2012), the original idea of Carol Barnes was not to use external reward or punishment to motivate animals to escape from a brightly lit platform. Thus, in the BM, the performance is not influenced by individual variability in hunger or thirst, unlike the radial arm maze. The BM is also less stressful for animals (especially mice) than the Morris water maze, because there is no water immersion and the subsequent high corticosterone increase which may affect the performance of animals (Harrison et al. 2009; Holmes et al. 2002; Pompl et al. 1999). In addition, BM is not so physically demanding for rodents than is the Morris water maze. The most often stated criticism of the BM task is that it is less sensitive to genetic alternations than is the Morris water maze. For instance, Stewart et al. (2011) expressed the view that the likelihood of detecting spatial memory impairments in Tg2576 mice (a model of Alzheimer’s disease) was the lowest when using the BM task (as compared to the T maze or Morris water maze). Vorhees et al. (2004) also found out that the BM task was less sensitive than the Morris water maze in detecting spatial learning and memory impairments in Sprague–Dawley rats exposed to 3,4-methylenedioxymetamphetamine; however, they concluded that presumably the test procedures used were not optimally configured to detect such impairments. Of note, there were comparative studies which indicated differences in effectiveness of the various mice and rat strains in the BM task (see below); thus, this should be taken account when choosing this task. Allied with this notion, Attar et al. (2013) indicate that when using this strain of mice (Tg2576), the used protocol should be rather shorter than longer because the differences in the performance are only seen when the less intensive training for this mouse strain is implemented. However, most reported papers assume that, generally, mice need longer and more intensive training in order to acquire the task. Still, many papers highlight the superiority of mice (especially C57BL/6J) to rats in the BM task studies. Mice are considered more efficient test subjects than rats due to their innate curiosity and desire to escape through small holes (Bach et al. 1995).

**Table 2:** The comparison of three commonly used tests measuring spatial learning and memory in rodents. For other comprehensive reviews, see Kapadia et al. (2016), Paul et al. (2009), and Vorhees and Williams (2014)

Feature (with comment if necessary)	Barnes maze	Morris water maze	Radial arm maze
Basic description of the task	An animal is placed in dry, circular platform and learns to reach target hole, located below the surface of the platform	An animal is placed in a circular pool and learns to locate a submerged platform	A food- or water-deprived animal is placed in the central hub and learns to obtain food or water from one of the arms
Recommendation regarding to species used	Mice > rats	Mice = rats	Mice = rats
Level of stress	Moderate	High	Moderate
Water immersion required	No	Yes	No (note: an aquatic version has been developed)
Physically demanding	Moderate	High	Moderate
Water or food deprivation required	No	No	Yes (note: animals should be equally motivated to find a reward)
Training length	Moderate	Moderate	Long
Odor cues (proper control of this confounding factor)	Yes	No	Yes
Measuring devices	Highly recommended	Mandatory	Highly recommended
Variations of the protocol to assess different aspects of spatial learning and memory	Yes (needs further validation, see “Variations of the protocol”)	Yes	Yes
Type of memory tested (depending on type of protocol)	Working memory; reference memory; cognitive flexibility	Working memory; reference memory; cognitive flexibility	working memory; working/reference memory

Beyond the aforementioned, there is a notion that the performance of animals in the BM task may be influenced by numerous non-cognitive factors (aromatic cues, anxiety, low exploratory activity, low motivation because there is no punishment). These factors, however, will not affect the behavior of the test animals if checked and properly controlled.

## Methodological considerations

### Apparatus

**Fig. 1:**
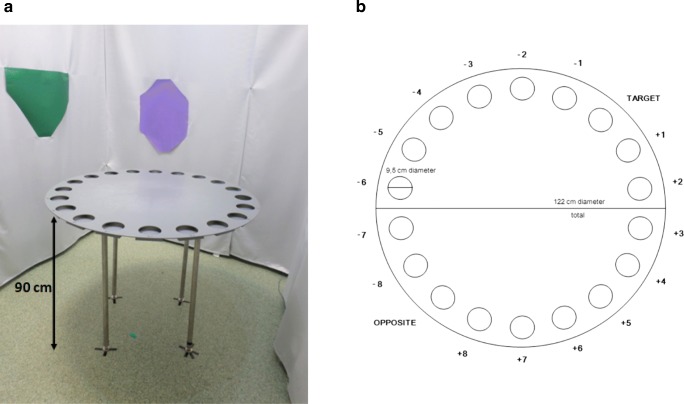
The room (**a**) and BM apparatus (**b**) used in our laboratory. **a** A total of four lamps are positioned above the maze, illuminating the platform’s surface evenly. Extra-maze cues in different colors are mounted on the white curtain backdrop surrounding the maze. **b** The dimensions of the BM apparatus to accommodate rats

The apparatus used in the BM task may have different parameters and may differ in platform material, color, and number of holes (see Fig. 1). Barnes (1979), in her original study, utilized a circular platform (122 cm in diameter) with 18 circular holes (9.5 cm in diameter) evenly spaced around the periphery. Moreover, the apparatus was located 91 cm above the floor to prevent the animals (rats) from jumping down. However, in the course of time, other platforms have appeared, and their size or color has varied from experiment to experiment, depending on the species used (mice or rats). To our knowledge, there is no paper in literature, either for rats or mice, regarding if and/or how the number of holes on platform circumference may affect their performance in the BM task. Nevertheless, in utilizing rats as the study species, the characteristics of the circular platform are usually as follows: 100–122 cm in diameter, with 18–20 circular holes, elevated 80–90 cm above the floor (Gawel et al. 2016; Greferath et al. 2000; Morel et al. 2015). Less frequently for rats, the number of holes around the circumference is 12 (Locklear and Kritzer, 2014; McLay et al. 1999). The platform size for mice is generally smaller (65–92 cm in diameter) than that for rats, but the number of holes around the circumference varies from 12 (Holmes et al. 2002), 16 (Bredy et al. 2004), 18 (Stragier et al. 2015) to 20 (Fowler et al. 2013; Kesby et al. 2015; Negrón-Oyarzo et al. 2015). When the size of maze for mice is 122 cm in diameter, the platform is usually pierced with 40 peripheral holes (Harloe et al. 2008; Raab et al. 2017; Ranney and Petro 2009). It should be understood that if the number of holes is 40, the acquisition (i.e., learning) of the spatial memory in mice usually needs more intensive/longer training, but the utilization of a 40-hole platform may reveal even very subtle differences between tested groups of animals because it seems to be more difficult.

With regard to the material, the platform may be made of solid wood, plywood, acrylic plastic, laminate, polyethylene, PVC, or metal. The color of the platform is usually white (O’Leary et al. 2011; Sunyer et al. 2007), rarely gray (Fowler et al. 2013; Gawel et al. 2016), or black (Morel et al. 2015; Vargas-López et al. 2011). However, the surface of the platform should be sufficiently neutral so as to ensure that the video-tracking program can distinguish the animal from the background. The platform material should also be easy to clean, should not change color under the influence of cleaning chemicals, and should not absorb odors.

In contrast to the color of the platform, the escape box located under one of the holes is often black. This choice of color serves as an additional motivation for the animal to escape from the surface of the platform due to the natural preference of rodents for dark environments. From the centre of the maze, all holes around the periphery are identical so that the animals cannot discriminate the escape hole from any other hole until they are situated adjacent to it. The remaining holes lead only to a false escape box, which, from the surface of the platform, appears indistinguishable. Usually, the escape box is made of the same material as the platform. Furthermore, it is selected randomly for each animal and in the classical BM protocol remains constant throughout the training sessions for a given animal, even though the surface of the platform is rotated between subsequent trials (O’Leary et al. 2011; Sunyer et al. 2007) in order to prevent odor cues from being used to resolve the maze.

There are two papers in which the BM apparatus had a different construction. In the paper of Koopmans et al. (2003), the authors expressed the opinion that mice do not prefer to drop down into a hole, thus, instead of peripheral holes, they used L-shaped exit tunnels turned 45° facing down with their open end. However, the authors clearly noticed that Swiss mice in this version of apparatus used instead of a spatial strategy, a thigmotactic strategy. Thus, on the basis of the paper of O’Leary and Brown (2012), this construction of apparatus should be rather avoided.

Youn et al. (2012) aimed to assess whether different reinforcements (aversive with fans or appetitive after food-deprivation) have an influence on performance of DBA/2J and C57BL/6J mice. For testing, they used a modified BM apparatus consisting of 4 quadrants with 2, 3, and 4 holes in the inner, middle and outer ring of each quadrant (in total 44 holes). This apparatus construction is believed to be more difficult for mice to resolve. Still, in comparing the performance of the two mice strains via this apparatus, they have found out that C57BL/6J mice did better only on the aversive version of the BM task, while in the appetitive version, both strains were equally competent. Such results seem to be very interesting but should be treated with some precaution because neither the classic BM apparatus nor the original ideas of Carol Barnes were employed.

Although there is no systemic study for what size the platform should be for the BM task in rats, most authors utilize the apparatus originally proposed by Barnes (1979). In this regard, the study of O’Leary and Brown (2012) deserves special attention because it may clarify this issue when mice are used as test subjects. In this work, the authors assessed the effect of apparatus design and test procedure (which will be discussed later) on the learning and memory performance of male and female C57BL/6J mice. Three different versions of the BM (but with the same 16 number of holes) were used: the small BM (69 cm in diameter), the small-walled BM (69 cm in diameter + wall around the edge of the maze), and the large BM (122 cm in diameter). In this experiment, the extended version of protocol was implemented: habituation, acquisition trials, acquisition probe trial, reversal trials, and reversal probe trial. During the experiment, the authors noticed that apparatus design did not influence the rate of acquisition learning, but it did affect the search strategy used by mice to find the escape box. They also revealed that with mice, adding a wall with intra-maze cues promoted the use of serial strategy (which does not rely on visuospatial cues), rather than the more efficient spatial search strategy (Harrison et al. 2006) (for the definitions, see Table 1 glossary). Likewise, the apparatus design had an influence on the reversal learning performance. In the small-walled version of the BM, since the reversal effect was not observed, the mice did not use visuospatial cues to locate the escape hole. However, the latency to find the escape hole, as well as the number of errors made, decreased with time when inside the small and large versions of the BM. In summary, the authors recommended the use of the large version of BM because there is a higher chance that mice will use spatial (but no serial or thigmotactic) strategy to resolve the maze. This was later confirmed by their follow-up study (O’Leary and Brown 2013).

Despite the obvious contribution of O’Leary and Brown (2012) for the optimization of BM apparatus design, it should be emphasized that in the paper of Sunyer et al. (2007), the most frequently adapted protocol using BM task in mice, the platform size was 92 cm in diameter, with 20 holes along the perimeter.

### Distant cues

Distant cues (extra-maze cues) are placed away from the edge of the platform at heights visible from the platform surface (see Fig. 1a). These can include gray drapery and partition screens (Barnes 1979), abundant visual cues (posters, colorful geometric shapes, black triangles, etc.) placed upon the walls of the testing room 50–200 cm distance from the edge of the maze, and visuospatial cues made of rigid black paper (e.g., circle or triangle) affixed to the walls. Of note, none of the cues are placed directly over an escape hole (O’Leary and Brown 2012). Additional cues may consist of objects within the room (McLay et al. 1998). In most BM experiments, the experimenter is usually not visible to the animal; however, the experimenter can also form an additional cue (Greferath et al. 2000; Ping et al. 2008). In such case, the experimenter should always wear something neutral in color, e.g., a white lab coat, and stay at the same place of environment, to avoid the situation in which the researcher inadvertently forms a new distal cue each run-through. Similarly, the distant cues should not be moved/changed between testing days—as a rule of thumb, the navigational reference points should always be at the same place in the environment. In the literature, there is no experiment which assesses how/if the number of cues may affect the performance of rodents. However, there are usually three–four colorful/black posters evenly spaced around the maze (Fowler et al. 2013; Kesby et al. 2015; Pompl et al. 1999; Sunyer et al. 2007; Youn et al. 2012). In our laboratory, for instance, apart from four different posters (yellow cross, red triangle, violet quadrant, orange circle) hung on the walls of the testing room, the laboratory surroundings (i.e., sink, table, door) were considered a part of environment. In one experiment, when Wistar rats were tested in this condition, we observed that the animals needed quite intensive training to acquire the task (4 days, 3 trials/day, in total 12 trials). When we hung a white curtain around the maze, and used the same four distant cues, the learning phase took a shorter time. Within these new conditions, we could observe that on the third trial of the day, the animals began to explore the maze instead of looking for safe shelter. It seems they already knew where the safe box was, yet, they did not reach it as quickly as in the first two trials. Thus, their performance and exploration of the maze were prolonged (i.e., primary latency) until they eventually reached it. Although we could clearly observe that they had acquired the task, the prevalence of clues increased the primary latency and the standard deviation of results were higher. Eventually, in the next set of experiments, we decided to decrease the number of trials to two/day but extend it 1 day (5 days, in total 10 trials). Thus, it seems that the number of cues within the surroundings of the maze affected the performance of rats in the BM task (Gawel et al. 2016; Marszalek-Grabska et al. 2018).

### Aversive stimulus

Although the BM task takes advantage of the natural preference of rodents for dark and quiet environments, weak aversive stimulation is often applied to provide inhospitable conditions and increase the motivation to escape from the platform, albeit, doing so without undue psychical or mental stress (Koopmans et al. 2003; Pompl et al. 1999). The open platform of the BM itself can be considered as an aversive stimulus, as rodents tend to avoid open spaces. Moreover, light sources (the most commonly used aversive stimuli in the BM task) can be affixed above the maze to brightly illuminate the platform (e.g., two 500-W floodlights) and motivate the animals to enter the dark escape box. However, when using bright illumination as a negative reinforcement to motivate animals, the source of light should be evenly placed above the surface of the maze. The open space, as well as bright illumination, creates an environment where behavior is motivated by rodents’ natural agoraphobia compelling a search among holes to locate a recessed goal chamber (Locklear and Kritzer 2014; McLay et al. 1998).

Other kinds of weak aversive stimulation applied in addition to bright light in order to increase the motivation to escape are buzzers or sound generators emitting tones of approximately 80–90 dB or rarely high speed fans (ideally, they should be suspended above the center of the platform). Although there are in the literature different approaches to adding buzzers as aversive stimuli, in most, a buzzer is switched on immediately when the start tube is raised and is not switched off until the mouse enters the escape hole (Bach et al. 1995; Gawel et al. 2016; Greferath et al. 2000; O’Leary and Brown 2012; Sunyer et al. 2007). While most authors used only bright light or a combination of bright light and buzzer as a negative stimuli, Inman-Wood et al. (2000) employed bright light, buzzer, and fan. With regard to this approach, such aversive stimuli (bright lights, fans, and sound) may, in reality, be distracting and can prevent the animals from focusing completely on the spatial task. This possibility should be especially taken into consideration when substances affecting attention processes are used. This was noted in the study by Inman-Wood et al. (2000), in which they assessed the effects of prenatal cocaine on the spatial learning and memory of offspring. Their results showed the existence of longer latencies during the first two trial blocks of the BM because the mice offspring were initially spending more time attending to aversive stimuli that were irrelevant to finding the goal. Nonetheless, once they acclimated, their performance was equivalent to that of controls.

### Measuring devices

Nowadays, as a measuring device, many researchers use a video camera suspended above the center of the maze and connected to a computer, allowing the observer to watch and record without disturbing the subject (e.g., Inman-Wood et al. 2000; Sunyer et al. 2007). Such an approach also facilitates data collecting, as a computerized recordable video camera system can enable a plotting of the track of the test animal. Hence, the computerized recordable video camera serves as a tool for contrasting the behavior of different animals, measuring total distance traveled or the time spent investigating escape and non-escape holes. One such system is Any-maze (Fowler et al. 2013; Harloe et al. 2008). With this digital tracking software, the latency to reach the target hole, distance traveled, and time spent in target quadrant can be measured. Another commonly used device is Ethovision, which enables measurement of escape latency, total distance traveled, number of errors, and the running speed of the animal (Koopmans et al. 2003). A modification of computer recording of animals in the BM was shown in McLay et al. (1999). The Opto-Varimex-Magnus animal monitoring system, which surrounds the platform, recorded the animal’s position, the distance the animal moved, and the amount of time the animal spent ambulating, resting and moving but not ambulating (twitching, making circular movements, etc.).

Despite the number of different available measuring devices, data may be collected by a highly trained observer with the use of manual stopwatches to record the latency to find the goal box, the number of errors committed by each animal, as well as the strategy used to resolve the maze (Harrison et al. 2006; Gawel et al. 2016). However, we strongly recommend prior to the BM task to test locomotor activity or motor coordination of animals (e.g., after the injection of tested compound) to assess whether potential disturbances are in fact memory impairments but not decreased locomotor activity. In this case, primary latency is not necessarily an appropriate parameter, because the animals are just slower. Other parameters (primary errors, search strategy, or score deviation) are more accurate parameters in such situations (for the definitions, see the Table 1 glossary). Nevertheless, when scoring the results manually, all experiments should be videotaped and later analyzed by experimenter blinded to treatment groups and strain of animals.

### Odor cues

It is important to note that after each BM trial, the platform surface and escape box are to be carefully wiped down in order to avoid the rodents utilizing olfactory cues to resolve the maze. For this task, most authors employ different dilutions of ethanol, ranging from 10- to 75% (Morel et al. 2015; Reiserer et al. 2007; Yassine et al. 2013). Ammonia-based cleaner, 30% isopropanol, water, water/vinegar solution, soap, Sparkleen, and water or 1% incidin solution are also used (Fox et al. 1999; Harloe et al. 2008; Pompl et al. 1999; Sunyer et al. 2007). In the original work of Barnes (1979), the surface of the platform was not cleaned, but only randomly shifted from trial to trial to avoid odor cues. Still, it is highly recommended to combine these two approaches when using the BM task, i.e., to wipe down the surface of the platform (using e.g., ethanol solution) and to shift the position of the platform randomly between individual animal trials. Because rodents have a common tendency to use odor cues in order to resolve the BM task, such approach will assure the experimenter that this confounding factor is excluded and does not affect the behavior of animal by giving false positive (i.e., memory impairments detection) results. Moreover, if the experimenter is in the same room that the experiment is conducted, regardless of whether he/she forms additional cue or is hidden, he/she should refrain from using any sort of scent. This is because the animals can easily notice this (Ranney and Petro 2009). Similarly, after the cleaning of the platform, all the contaminated cleaning materials should be immediately removed from the testing room. When using ethanol for cleaning, the concentration of ethanol vapors in the air could reach a level which is anxiolytic and which influences the performance of the animals; thus, a proper ventilation of testing room is mandatory.

## BM procedures

The BM task is customizable to many experimental conditions, depending on the specific aim of the experiment. Although there are a variety of published methods for running this task, it is worth reviewing the basic principles and phases of the BM task, as well as the most frequently adopted protocols. It is also worth mentioning papers which have definitely contributed to the methodological improvement of the BM protocol. It is, thus, worthwhile to first become familiar with Carol Barnes’s original description (1979), because most of the protocol was later adopted by other researchers. In this study, a proper acquisition phase was preceded by a single 4-min long habituation session in which the rat was placed directly in the escape box. Subsequently, the rat was returned to its home cage for 1 min, after which the training phase started. Each rat received two trials per day for 6 days—each with a 1-min inter-trial period. At the beginning of each training trial, the rat was gently placed in the middle of the maze and the start chamber was placed over it. After a 30-s delay, the chamber was raised and the trial begun. The animal was allowed to explore the maze for up to 4 min. If the rat found the escape box before 4 min had elapsed, it could explore the escape chamber for 1 min before being returned to its home cage. If the rat did not find the safe shelter within 4 min, it was placed inside it by the experimenter, and after a delay of 1 min, it was returned to its home cage. The position of the safe shelter remained constant from trial 1 until trial 7. On trial 8, the safe chamber was rotated 135° from its original position and the new positioning kept constant until trial 11. Although Carol Barnes noted that this methodology was not exactly a reversal learning trial, this term was adopted for all trials in which the position of the escape hole was changed from the initial.

### Habituation (i.e., shaping trial)

Whether the test subjects are mice or rats, in order to familiarize the animal with the maze and to reduce the levels of anxiety which may affect the behavior of the animal (i.e., locomotor activity) during the first few learning trials, the animals must first be habituated to the escape box and/or platform, prior to the first acquisition trial. Usually, this habituation is done 24 h before, but sometimes, this comes about immediately before the first acquisition trial begins. With regard to habituation trial practice, some authors put forward one shorter (1–2 min) session that is only within the escape box (Fox et al. 1998; Greferath et al. 2000), while others suggest a longer session (4–9 min) in which escape box and platform exploration is allowed (Locklear and Kritzer 2014; McLay et al. 1999; Rodriguez et al. 2013; Vargas-López et al. 2011). In the original experimental protocol, Barnes (1979) placed the rat directly in the escape box (4 min), the rat was then removed, and after a 1-min inter-trial interval, the first acquisition trial occurred. In our laboratory, we first habituate the animal to the maze for 1 min during which the rat is allowed to freely explore it, and then we gently guided it to the escape hole for another 2 min. This is done 24 h before the first acquisition trial. We believe that this type of habituation, preceded by the handling of the animals for few days prior the BM task, is enough for the rats to familiarize themselves with both the maze surface and escape box, as well as to reduce their anxiety (Gawel et al. 2016; Marszalek-Grabska et al. 2018). In the Sunyer et al. (2007) work (the most frequently adopted procedure for the BM task in mice), the authors recommend one session (the shaping trial) in which they placed the mouse in the center of the maze and covered it by the chamber, lifting this 10 s later. Subsequently, the mouse was gently guided to the escape hole and allowed to stay there for 2 min. Immediately following the shaping trial, the first acquisition trial begins.

Because of uncertainty as to how and to what extent the test procedure affects the learning and memory performance of C57BL/6J mice, in the study of O’Leary and Brown (2012), during the shaping phase, half of the tested mice received one habituation trial, while the other half underwent four trials. In both cases, the mice were placed underneath a transparent 2-L beaker and they could explore the escape hole and its close surroundings for 5 min. In addition, they could view the entire the maze environment. After testing, the authors noticed that the number of shaping trials did not affect the overall learning and memory performance of the mice, even though they clearly stated that the mice were extensively handled and also tested before the BM task. They held that presumably this approach reduced the anxiety of mice in the BM task, but they hold that four habituation trials be conducted if the test animal’s anxiety levels or motor abilities are unknown.

In standard testing, during the habituation trial conducted 24 h before the first acquisition trial, the source of aversive stimuli (such as buzzers) is switched off, while the bright lights are usually switched on. Moreover, the extra maze cues are blocked so as to ensure that latent learning will not occur (O’Leary and Brown 2012; Stouffer and Heisey 2013). Alternatively, during the habituation, the experimental room may be lit using red bulbs (Vargas-López et al. 2011).

### Acquisition trials

With regard to the acquisition phase, the duration of the training trial, as well as the number of trials per day, may vary from study to study, but it is notable that mice need more time to acquire the task than do rats. The standard procedure is as follows.

At the beginning of each acquisition trial, the animal (mice or rat) is placed in the middle of the maze and is covered with the start box which may be a cylindrical start chamber, an opaque tube or a bucket (e.g., Barnes 1979; Kesby et al. 2016; McLay et al. 1999; O’Leary and Brown 2012). After a delay of several seconds (10–15 s), the start box is lifted and the rodent can then explore the platform. The intent of this approach is to ensure that the initial orientation of the animal in the maze varies randomly from trial to trial (similarly to different starting positions in the Morris water maze). For the rats, the usual experimental setup consists of two trials per day for 5 days, up to 2 or 3 min duration (Chawla et al. 2017; Maegele et al. 2005; Stouffer and Heisey 2013; Walesiuk and Braszko 2010). Typically for the mice, as proposed by Sunyer et al. (2007), the acquisition phase consists of four sessions per day for four consecutive days. In contrast, O’Leary and Brown (2013) hold that the number of trials in the acquisition phase affect the performance of mice and recommend two trials/day, for 15 days, up to 5 min each trial. Moreover, they suggest that the training phase should consist of at least 24 trials. In their opinion, it is only then the mice demonstrate reliable use of spatial search strategy. Note: it is very difficult to explain why there are discrepancies in these two methods, because both authors used very similar experimental conditions, as well as the same strain of mice (C57BL/6J).

In standard testing, during the acquisition trials, the animals are allowed to freely explore the maze and using the distal cues, to localize and to enter the escape box. If the animal does not enter the escape hole up to a pre-determined amount of time, it is gently pulled by the experimenter to the escape box and is allowed to stay there for 30–60 s before being return to home cage. In a setup with few trials per day, independently of species used, a typical inter-interval is app. 15–20 min (Gawel et al. 2016; Locklear and Kritzer 2014; McLay et al. 1999; Negrón-Oyarzo et al. 2015). While mass trials with 1 min inter-trial intervals have been described in the BM task (Harrison et al. 2009; Yassine et al. 2013), spaced trials with longer inter-trial intervals are considered to enhance performance in spatial memory tasks (Kapadia et al. 2016). As a rule of thumb, the groups should run in alternating fashion, i.e., control–experimental–control.

### Acquisition probe trial(s)

The probe trial (sometimes called the transfer test) allows ascertaining whether the trained animals use the distal cues to create a spatial map of their environment and are able to locate the hole which previously contained the escape chamber. In other words, the measure of the spatial bias is where the animal spends its time during this phase. During this phase, the target hole is closed and the time spend on the vicinity of the previously correct hole (or the correct zone) is measured. Usually, independently of the used animal species, the duration of probe trial is 90 s (Gawel et al. 2016; Rodriguez et al. 2013; Sunyer et al. 2007), 120 (Morel et al. 2015), or 180 s (Harloe et al. 2008; Sunyer et al. 2007). Irrespective of the length of the acquisition probe trial, this is in harmony with the observation of O’Leary and Brown (2012), who indicate that a probe trial longer than 3.5 min may underestimate the spatial memory of mice because after this time the performance of mice is more variable (the mice already know that there is no escape box at the previously known spot and they have started exploring the other parts of the maze).

When the probe trial is conducted shortly after the last training trial, it is difficult to clearly state whether the test animal is utilizing reference or working memory (Vorhees and Williams 2014). Thus, there is a prevailing approach to do it 24 h after the last acquisition learning trial (Harloe et al. 2008; Kesby et al. 2015; Morel et al. 2015). This is because this time lapse assures the researcher that spatial reference memory (short term) is being tested. In certain experiments, the second probe trial takes place after an interval of 7 or 10 days. In this regimen, long-term memory retention (reference memory) is being assessed. Such testing may be followed by reversal learning trials or extinction trials.

When extinction trials are applied, after the animal acquires the BM task, it is exposed to the paradigm for a few days during which the escape box is no longer present (Harloe et al. 2008; Vargas-López et al. 2011). This procedure allows to assess how quickly the test animals learn that safe shelter is no longer available and there is no way to escape from the maze.

After a few trials, the time spent in the zone which previously contained an escape hole decreases substantially, while the exploration of other zones increases. Harloe et al. (2008), through their experiments with rimonabant (as endocannabinoid CB1 receptor antagonist) which impairs the extinction learning in the aversive version (bright light and fans) of the BM task, conclude that this type of BM protocol may be useful for investigating the extinction of behaviors associated with aversive memories, e.g., in an animal model of post-traumatic stress disorder.

### Reversal learning trails

The reversal learning trials usually start 24 h after the last acquisition trial or acquisition probe trial(s) (Bredy et al. 2004; O’Leary and Brown 2012) and take shorter times than that in the acquisition phase (Raab et al. 2017; Stragier et al. 2015; Marszalek-Grabska et al. 2018). This is because the animals have already familiarized themselves with the environment (e.g., distant cues), and they know the rules of how to solve the maze. In the reversal learning phase, the escape shelter position is changed (e.g., 120°, 135°, or 180°) from its previous location in the acquisition phase. Under these new conditions, the animals have to learn that the former is no longer rewarded and they have to switch their strategy in order to resolve the maze. This also allows researchers to detect whether the animals really use extra-maze cues to locate the safe shelter (O’Leary and Brown 2013). The re-learning of new positions usually occurs faster than in the acquisition phase, and, if an improvement in performance is not observed within the reversal phase, this means that cognitive flexibility is disturbed (Barnes, 1979).

The reversal learning phase may be followed by a reversal learning probe. This allows an assessment, if done 24 h or 7 days after the last reversal learning trial, of the short-term or long-term memory retrieval of new safe shelter position (Bredy et al. 2004; Chawla et al. 2017; O’Leary and Brown 2012). In such trials, the procedure parameters (e.g., time of trial, inter-trial interval) are the same as in the acquisition phase.

### Dependent measures and search strategies

Technological development has allowed a measuring of additional parameters, e.g., distance traveled or speed (for the definitions, see Table 1 glossary). Such parameters must be controlled because the time needed to reach a target hole may be due to the test animals being slower, and not because they are spatial learning impaired. What is more, during the acquisition of a BM task, the rodents sometimes lack motivation. This is especially evident after a few learning trials, because animals have already familiarized themselves with the environment and the aversive stimuli. When starting a new trial, they will directly go to the escape hole, put their heads to the escape hole to assure themselves that it is still there and instead of entering into it, they again explore the maze. Thus, this practice increases the latency to enter the escape hole, as well as the number of committed errors due to their further exploration of the maze. This can engender misinterpretation. Thus, Harrison et al. (2006) calculated latency (primary latency), path length (primary path length) and number of errors (primary number of errors) only to the first encounter with the escape hole. This approach was subsequently employed by others (Locklear and Kritzer 2014; Rodriguez et al. 2013; Sunyer et al. 2007), as well as by our laboratory (Gawel et al. 2016; Marszalek-Grabska et al. 2018). Moreover, O’Leary and Brown (2013) also noticed that primary measures are more sensitive in terms of learning/memory impairments than total measures.

**Fig. 2:**
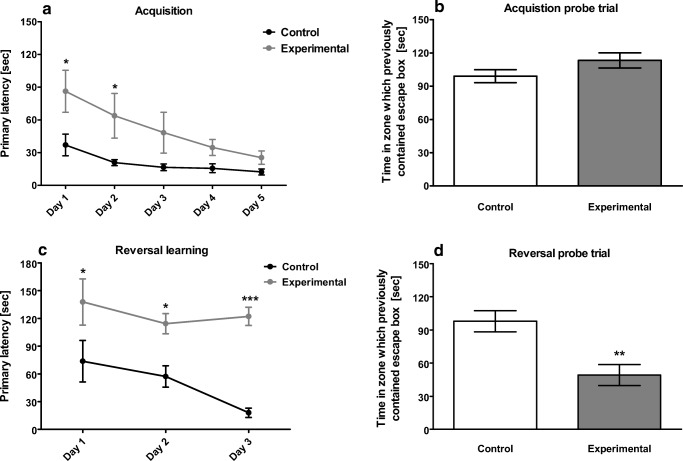
An example of BM data. At first glance, one may assume that the study reveals spatial memory impairments in experimental group (**a**) alone, due to statistical differences in primary latency between groups in 1st and 2nd days of learning. In fact, both groups acquired the task, but the experimental group needed a longer time to do so (steeper slope). This effect was confirmed in the probe trial (**b**), conducted 24 h after the last training trial. Both groups spent similar amounts of time in the zone (one fourth of the maze) which previously contained the escape box. However, when the reversal learning trials were conducted, the animals in the experimental group did not re-learn the new position of the safe shelter (**c**). This was confirmed in the reversal learning probe trial, when the animals spent less time in the zone providing a new position of safe shelter, compared to the control group (**d**). In summary, the substance tested did not affect spatial memory acquisition, but impaired animal cognitive flexibility

O’Leary and Brown (2013) aimed to clarify what measures of performance are the most sensitive for detecting memory impairments during acquisition/reversal trials, and concluded that latency to escape hole was the least sensitive parameter for detecting learning impairments. Instead, they indicated that the number of primary errors, the hole deviation score, and the distance traveled are the most sensitive measure of animal performance, and, hence, are the better markers (for an example of data, see Fig. 2).

During the acquisition probe trial and/or reversal probe test, some authors measured only the time to reach the target hole (which was blocked during this test) (Gawel et al. 2016; Morel et al. 2015), while others assessed the time spent by the test animal in the zone in which, previously, the escape box was located (Harrison et al. 2006; Koopmans et al. 2003; Stragier et al. 2015). Sunyer et al. (2007) proposed to calculate latency and path length to reach the virtual target hole, as well as the number of pokes per each hole. This is in contrast to the conclusions of O’Leary and Brown (2012, 2013), who think that the percent of time in the correct zone or the proximity to the escape hole is a more sensitive measure of performance in the probe trial than is the number of nose pokes. Of note, most frequently, in such studies, the escape hole or escape hole + two adjacent holes are defined as a zone-of-interest (Stragier et al. 2015).

Despite the parameters discussed above, when conducting the BM task, the collection of search strategies using by animals is highly recommended (for a summary of the definitions see Table 1). This is because the analysis of this data may clarify whether the animals actually use spatial strategy to resolve the maze. For example, if aged animals are used, the time to reach a target hole may be prolonged due to physical deficits. In such case, the analysis of search strategy may give additional insight whether they exhibit spatial learning impairment. There are three different strategies used by animals to resolve the maze. When the spatial (i.e., direct) strategy is used, animals move directly to the target hole or 1–2 adjacent hole(s) before visiting the target. This strategy in non-cognitively impaired animals is usually observed after a few consecutive trials and suggests the fact that animals use spatial clues to resolve the BM task. When the serial strategy is used, the animals visit serially the adjacent holes in a clockwise or counter clockwise manner. Although they can reach the target hole as fast as the control group, this does mean that they do not use spatial clues to resolve the maze. A subtype of serial strategy is thigmotaxis, when animals move along the wall in the periphery of the platform. Thus, to avoid this type of strategy, the use of walls along the periphery of the BM platform is contraindicated. The third strategy used by animals is a mixed (i.e., random) strategy when animals searching the holes cross the maze through its center or investigate the holes in an unsystematic fashion. Indeed, when after a few days of learning trials the animals still use this strategy to resolve the maze, it means that they are cognitively impaired and do not employ spatial clues to reach the target hole (O’Leary and Brown 2009; Sunyer et al. 2007; Yassine et al. 2013).

### Variations of the protocol

Although the BM task is commonly used to measure spatial learning and reference memory (both short and/or long term), there are few variations of the original protocol that have been designed to test working memory (i.e., the matching-to-sample-test) or non-cognitive deficits (i.e., the cued version). In order to assess working memory (or short-term memory), commonly in the BM task, test animals were given 4 (Fedorova et al. 2007), 5 (Livingston-Thomas et al. 2015) or 10 trials (Ryan and Vandenbergh 2006) per day. Herein, the position of the escape hole was held constant throughout all trials per 1 day but changed over to another random position at the first trial on the following day. Thus, the animals were forced to re-learn the location of the escape hole every day. This is a memory activity which is believed to be dependent on the function of the prefrontal cortex (Livingston-Thomas et al. 2015). The first trial each day was thus a simple trial because the animals were exposed to the new location of the escape hole. Each subsequent session, following a 1-min delay (Livingston-Thomas et al. 2015; Ryan and Vandenbergh 2006) or a 15-min delay (Fedorova et al. 2007), was a matching-trial because the animals were re-tested with the escape hole in the same position as in the first trial of that day. Although this protocol seems to be very interesting, it needs further investigation so as to understand whether and to what extent the inter-trial interval can affect the efficiency of animals in re-learning the new location in the BM task. Similarly, there are inconsistencies in terms of days of learning (in total 15, 24, or 50 trials).

The cued version of the BM has also been described in the literature. Briefly, the cued version may clarify if the memory impairments detected in the spatial version of the BM task are what they supposed to be and are not caused by lack of motivation, anxiety, or visual disturbances. In this variation of the protocol, a discrete visible cue (e.g., a small colorful object like tube, can or cone) is placed directly behind the hole containing the escape box (Bach et al. 1995; Fox et al. 1998; Paylor et al. 2001; Radyushkin et al. 2005; Ranney and Petro 2009) or is attached to the maze perimeter adjacent to the escape box (Reiserer et al. 2007). The position of the escape hole varies from to trial to trial or from day to day (if the mice are tested once daily). This version of BM does not require hippocampal but rather striatum function, because the animals need to associate the proximal cue with the position of the escape box.

While some authors conduct the cued version after the spatial (Paylor et al. 2001), others conduct the cued version first (Ranney and Petro 2009; Radyushkin et al. 2005). The order of the experiments may actually have an influence on the performance of the test animals. In the paper of Reiserer et al. (2007), some of the mice received the cued version before the spatial, while some had the spatial before the cued. In such study, the test mice showed impairment on the spatial BM version task when they were pre-exposed via the cued version. When the cued version followed the spatial, such impairment was not seen. Thus, Reiserer et al. (2007) conclude that other cognitive or non-cognitive processes masked deficits in the traditional version of the task. Nevertheless, further investigation is needed as to how/why the version order affects animal performance in the BM task.

Rarely described in BM practices, the implementation of the time-to-reach-criterion allows to quickly measure the ability of animals to learn escape box location. In this protocol, the animals are trained until they reach specific criterion. Unfortunately, there are inconsistencies in literature in terms of definition of this criterion. For instance, Bach et al. (1995) and Inman-Wood et al. (2000) defined this criterion as seven out of eight trials with three or fewer errors, Ping et al. (2008) do so with two or less errors, while Holmes et al. (2002) establish more restrictive criterion (less than one error). In contrast, Coleman et al. (2014) defined it as less than 20 s average latency to escape hole. We ourselves hold that the criterion based on the errors is more accurate than that based on the time. In fact, days per criterion might be a useful BM parameter, especially in studies of aged animals (Rosenfeld and Ferguson 2014; Barrett et al. 2009; Greferath et al. 2000).

### Interpretation of data—an example

Figure 2 exemplifies the data obtained after a BM run. As seen in Fig. 2a, the experimental animals took longer to reach the target hole at the 1st and 2nd days of acquisition, in comparison to the control group (two-way analysis of variance with repeated measures, group × time interaction). Although at the 3rd day of the learning phase, the animals still needed more time to locate the target hole, the results are no longer statistically significant. Moreover, there is no difference between tested groups in the primary latency on the next 2 days of learning. At first glance, one may assume that experimental groups are cognitively impaired (steeper slope of leaning), but they eventually acquired the task. Indeed, when the acquisition probe trial was done 24 h after the last learning session, the animals of both groups spent a similar amount of time in the zone which previously contained the target hole (see Fig. 2b) (note: during acquisition probe, the escape to target was blocked). However, when the reversal learning trials were conducted, the animals in the experimental group did not re-learn the new position of the safe shelter (see Fig. 2c). In contrast, in the course of reversal learning trails, the control group did successfully re-learn the new position of the escape hole which was evident at the 3rd day of the reversal leaning phase. When the reversal probe trial was conducted (see Fig. 2d), analysis of data revealed that the experimental group spent less time in comparison to the control group in the zone which previously contained the new position of safe shelter (during the reversal probe, the escape to target was blocked). In conclusion, both groups of animals have learnt how to resolve the maze and acquire the task; however, only the control group was able to re-learn a new position of safe shelter and did not exhibit cognitive flexibility impairment.

## Test subjects

First and foremost, the choice of animals is a critical and independent factor when using the BM task. In fact, the choice of test species (whether rat or mouse) and strain of animals should be taken into account when planning the experimental design. This is because the difference in the genetic background of diverse strains (especially mice) makes them not equally effective in solving the maze. For instance, if the animals show high level of anxiety, they may spend the time on the surface of the maze not actively looking for the escape box, but just grooming or freezing. In contrast, when the chosen strain shows low levels of anxiety, they may explore the maze rather than looking for the safe shelter. In both cases, the time to reach the escape box may be prolonged and not truly reflect memory impairments.

### Strain

In many studies, genetically modified animals are used as models of the neurodevelopmental and neurodegenerative disorders associated with learning and memory deficits. The BM task was first used with mice by Bach et al. (1995) to assess learning and memory in the calcium/calmodulin-dependant protein kinase II mutant mouse. O’Leary and Brown (2012) note that since then, BM has been used to assess visuospatial learning and memory in different inbred mouse strains (Koopmans et al. 2003; O’Leary et al. 2011). Examples include transgenic mouse models of Alzheimer’s disease (Pompl et al. 1999; O’Leary and Brown 2009), the FMR mouse model of Fragile-X disorder (Yan et al. 2004), and mice that have targeted mutations within genes implicated in learning and memory (Zhang et al. 2002; Seeger et al. 2004; Rizk et al. 2004). Various strains will show differences in learning, memory ability and synaptic plasticity. This was seen by O’Leary et al. (2011) who, using the BM task, examined strain and sex differences in the learning, memory and search strategies of 13 inbred mouse strains with different visual abilities. In doing this, they reported significant differences between inbred strains in their performance during acquisition and reversal training. In brief, the results showed that C57BL/6J and CAST/EiJ mice achieved better primary latency times and made less primary errors than did other strains. That C57BL/6J mice hold better learning performance has also been recognized via experiments utilizing the Morris water maze (Brown and Wong 2007; Patil et al. 2009). This suggests, therefore, that this strain has superior spatial learning ability. Brown and Wong (2007) also note that A/J and 129S1/SvImJ mice took the longest of all strains to locate the escape hole in the acquisition and reversal learning phases. Long latencies of these strains can be explained by their slow movement when exploring the maze and the amount of time they spend being immobile. Interestingly, the performance of A/J mice does not improve, suggesting that this strain has impaired learning in the BM task. A trial that was conducted by Koopmans et al. (2003) tested spatial learning capacity in another, widely used strain of mice. The results support previous reports that C57BL mice are suitable for testing spatial learning and confirm the poor performance of BALB/c and Swiss mice. Thus, the data of Koopmans et al. (2003) questions the ability of these two strains to acquire spatial tasks. Nguyen et al. (2000), as well as Koopmans et al. (2003), have also reported strain differences in spatial abilities. Herein, the variation between strains as seen in the long-term retention phase is considered to be due to genetic background and experimental task (Nguyen et al. 2000; Yoshida et al. 2001).

In rat study literature to date, there is no comparison of different strain performance in BM-based experimentation, although studies comparing spatial learning abilities of rats indicate the good performance of Wistar rats. These outperform Sprague–Dawley and Long–Evans rats, even though albino Wistar rats are known to have worse visual functions than pigmented rats (including lower visual acuity and impaired vision). Such findings indicate that in small rodents, high visual acuity is not required for successful performance in visuospatial tasks where the visual cues are at a distance of 1.5–2 m (Gökçek-Saraç et al. 2015). The most popular strains used for BM are Wistar rats (Gawel et al. 2016; Trofimiuk and Braszko 2011; Vargas-Lopez et al. 2011) and Sprague–Dawley rats (McAteer et al. 2016; Locklear and Kritzer 2014). In Komater et al. (2005), Long–Evans rats have also been used, while Greferath et al. (2000) performed trials on Dark Agouti rats.

In summary, strain-related differences in animal performance in cognitive tasks cannot be underestimated since such differences may affect the outcomes of the experiment (Gökçek-Saraç et al. 2015).

### Sex

Recent studies have shown that estradiol, the female gonadal hormone, enhances learning and memory tasks, both in humans and animals. However, the processes and neural systems involved in the effects produced by estradiol are currently unknown. Interestingly, the majority of studies in rats and mice did not find a clear gender difference in spatial learning, but the studies which did find a gender difference indicated that male rats and mice (DBA/2J and C3H/HeJ strains) performed better than females in the BM (Barrett et al. 2009; O’Leary and Brown 2009; O’Leary et al. 2011). These observations have also been confirmed in experiments using a modified BM test. Herein, males show better memory performance and larger reversal effects than did females during reversal training (O’Leary and Brown 2009, 2012). It must be noted that these data were not replicated subsequently for reversal effects, due to the high variability in the size of the reversal effects seen in female mice. The observed effect seems to be related to sex differences in used search strategy. A growing body of evidence suggests that the males appear to learn to employ the spatial strategy more rapidly and effectively than do females, resulting in a better probe trial performance, and causing males to persevere longer than females at the original escape hole during reversal trials (O’Leary and Brown 2012).

The differences in behavior between genders may also be due to several non-mnemonic factors which include animal age (Bimonte-Nelson et al. 2003; Kanit et al. 2000), hormone status (Bimonte and Denenberg 1999; Galea et al. 2001; Goudsmit et al. 1990), differences in apparatus design, the procedure employed across the studies (Berger-Sweeney et al. 1995; Roof and Stein 1999), and the strain of mouse used (O’Leary et al. 2011). Furthermore, the estrous cycle is recognized as a strong determinant in emotionality and the cognitive capacity of female rodents. This may result in morphological changes in the brain that are observed as an increase in spine density in the CA1 region of the hippocampus especially during proestrus, and then as a decrease in the estrus phase and further seen as an intermediate spine density in the diestrus (Woolley et al. 1990; Woolley and McEwen 1992). It should be underlined that in behavioral/hormonal studies, females are also of interest due to the cessation of ovarian function in both aging humans (menopause) and rats (estropause) with related sequelae.

A few previous studies have shown the influence of testicular hormones on spatial memory in rats (Gibbs 2005). Indeed, Kritzer et al. (2007) show that castration influences the performance of prefrontal cortex-dependent memory tasks and that both testosterone and estradiol contribute to this effect. Still, some researchers have noted that sex differences can be observed only in limited test phases. No sex differences were noted during the acquisition training, but they were presented as bringing about a larger reversal effect for males than for females. These differences may be due to the perseverative search strategy used by male mice, as opposed to that employed by females. An increased tendency for males to search at the previous escape hole location was also found by O’Leary and Brown (2009). This strategy is probably not related to the more holding of an accurate spatial memory for the escape hole location since no sex differences in probe trial performance were observed. However, the sex differences in the reversal effects may be a product of spatial perseveration in males or increased stress in females produced by the aversive stimuli used during training, as such effect would discourage perseverative visits to a hole that did not provide escape (Locklear and Kritzer 2014; O’Leary and Brown 2012). The differences between the sexes in rats are evident not only in the level of hormones but also in basal and stress-induced corticosterone concentrations. These are higher in females (Carey et al. 1995; Figueiredo et al. 2002). Moreover, stress seems to affect spatial learning differently in males and females. Indeed, researchers have observed that in response to acute and chronic stress, spatial learning and memory are impaired in male rats in several spatial tasks such as the radial arm maze, Y maze, or Morris water maze. However, very few differences have been reported in the BM test. In standard conditions, the preferred strategy of male mice and rats is spatial (Bettis and Jacobs 2009; Schwabe et al. 2008, 2010; Tropp and Markus 2001). Chronic and acute stress, however, induce a switch from spatial towards a stimulus–response strategy (Schwabe et al. 2008, 2010) in male mice. In contrast, females apply both spatial and stimulus–response strategies (Bettis and Jacobs 2009; Korol et al. 2004; Pleil and Williams 2010; Tropp and Markus 2001) with preference for the spatial strategy during proestrus (Korol et al. 2004; Pleil and Williams 2010) and acute stress. Females are also considered more sensitive to the effect of stress, depending on the duration, the type of stressor, and the spatial task*.*

### Aging

In humans and rats, age is considered a crucial factor in cognition, bringing about a decline. In addition, it influences the morphology of brain structures involved in cognition processes. In females, changes during aging may be also potentiated by the loss of gonadal hormones. The effect of aging on spatial memory and learning in rats and mice has been intensively studied using BM. Barnes (1979), for example, reported that senescent Long–Evans rats (28–34 months) were impaired relative to younger rats (10–16 months) on all of the dependent measures. Moreover, the older rats were notably impaired on reversal trials in which they were required to learn the new localization of the escape box. Similar behavior was observed in aged Fisher 344 (Barnes et al. 1991), Sprague–Dawley (Barrett et al. 2009), Dark Agouti (Barrett et al. 2009), and Fisher 344 × Brown Norway rats (McLay et al. 1999). During all experiments, older rats show impairment on all dependent measures of the BM, including latency to reach the escape box, the number of errors made, as well as speed and distance covered (Barnes 1979; Barnes et al. 1980; McLay et al. 1999). Furthermore, researchers observed that the motivation to search for the escape hole declined with age, albeit without decline of locomotor activity (Morel et al. 2015). While several studies have indicated that the progressive decline in spatial memory performance in aged rats is associated with cellular and molecular changes in the hippocampus, most of the reported data has been collected from males alone (Begega et al. 2012; Geinisman et al. 2004; Jacobson et al. 2008; Sugaya et al. 1996).

Similar cognition deficits have also been reported in different strains of mice (King and Arendash 2002; Koopmans et al. 2003; Moffat and Resnick 2002; Patil et al. 2009). Indeed, Bach et al. (1999) saw that aged C57BL/6 mice made more errors than did the young (3 and 6 months), and they more often used a serial search strategy rather than a spatial strategy. These results are contrary to that observed for rats (Barnes 1979), which were able to switch strategy to spatial. The development of gene targeting technology has enabled the generation of gene mutations in rodents, especially mice. This increased interest in use of mutant mouse models has allowed an evaluation of the relationship between the aging and cognition, e.g., as a model of Alzheimer disease. Stewart et al. (2011) have indicated that in the tg2576 mice (model of Alzheimer’s disease), deficits in spatial memory are observable at 3–7 months and at the age when amyloid deposition occurs (8 months and older). This high variability in the age of beginning of cognition deficits may be related to the development of deep visual impairments in individuals. Therefore, BM has been used to test spatial memory in mouse models bred on genetic backgrounds carrying mutations associated with different degrees of visual impairment (e.g., in SLJ, C3H/HeJ, CBA/J, FVB/N backgrounds) (Yassine et al. 2013). Yet, performance differences between inbred strains on the BM are not always replicable across studies (Holmes et al. 2002; Nguyen et al. 2002; O’Leary et al. 2011), and on some designs, blind mice can perform as well as sighted mice can, indicating that visual cues are not being used (Garcia et al. 2004; O’Leary et al. 2011; Yassine et al. 2013). These problems may be due to the use of suboptimal apparatus designs or procedures in the testing of mice within the BM. Moreover, in the course of research on the behavior of aging rodents, it was noted that training was able to decrease this deficit and to lead aged mice to reached a plateau comparable with the young by day 4 in most parameters (Barreto et al. 2010). Therefore, the BM test is useful in assessing outcome in neurodegenerative disease and postoperative dysfunction of cognition (as carried out in aged mice).

## An extended BM protocol

Researchers should follow all the basic steps set out by Carol Barnes, because differences between the groups might be seen in the reversal trials and not in the acquisition phase (Coleman et al. 2014; Fowler et al. 2013; Marszalek-Grabska et al. 2018). Thus, herein, based on the original work of Carol Barnes, we propose an extended protocol of BM for rats which may be customized for mice also, and which is built upon on literature data, our previous papers (Gawel et al. 2016; Marszalek-Grabska et al. 2018), as well as our own observations and experience. The aversive stimuli and room configuration proposed here have been validated in our laboratory.

### Preparation of the room and computer setup (if applicable)

The testing room is prepared as follows: (1) a light mono-colored curtain (e.g., white or light gray) is hung around the circular platform approximately 100–150 cm from the edge of apparatus; (2) four visible cues (yellow cross, red triangle, violet quadrant, orange circle) are placed evenly on the curtain approximately 15 cm above the level of the surface; (3) none of the visible cues are placed directly behind the escape hole; (4) no source of odor is permitted in the testing room (e.g., trash bin); (5) the buzzer (80–85 dB) is prepared; (6) two lamps, 150 W each, are affixed directly above (1.5 m) the surface of the platform; (7) the computer and tracking software are switched on, and parameters are setup as recommended; (8) as bright light may bounce off the platform’s surface and interfere with the quality of video, to curtail this effect, the light source is to be adjusted before the experiment starts.

### Phases

The protocol described below is a classical but extended BM protocol which consists of five phases: (1) habituation, (2) acquisition phase (i.e., learning), (3) acquisition probe trial (24 h after last training session: short-term reference memory), (4) reversal learning trials (cognitive flexibility), and (5) reversal learning probe (24 h after the last reversal learning trial: short-term memory retrieval of new escape hole position). The animals should be transferred into the testing room at least 30 min before the experiment begins, in order to for them to acclimate to the environment.

During the acquisition phase and reversal learning trials, the following parameters are to be calculated: (1) primary latency, (2) primary errors, (3) hole deviation score, (4) strategy (spatial, serial or random), (5) distance traveled (in cm), and (6) speed (cm/s). If tracking software is not available, the first three parameters are to be measured. In such situation, an experimenter blinded to experimental groups and strain of animals should re-analyzed the video files independently.

During the acquisition probe test and reversal learning probe test, the following parameters are to be calculated: (1) time in the correct zone, (2) primary latency, and (3) primary errors to reach the previously target hole.

### Habituation (day 0)

1. Twenty-four hours before the acquisition phase, in order to reduce anxiety behavior, the rat should be habituated to the platform and escape box. In doing this, the rat is allowed to explore the maze freely for 1 min and then is put gently into the escape hole. The hole box is then covered, and the animal stays there for 2 min before being returned to their home cage.
2. The lights are switched on, but the buzzer is switched off. Distant cues are also blocked.

#### Acquisition phase (days 1–5)

1. The rat is removed from its cage and gently put in the center of the maze. The animal is immediately covered (using, e.g., a box), and it is left under the cover for 15 s. The box is then lifted, the buzzer is switched on, and recording begins.
2. The rat is permitted to explore the maze and the holes until it locates the escape hole. If the rat enters the escape hole, it is covered and the rat is allowed to stay there for 30 s. During this time, the buzzer is switched off. If the rat does not enter the escape hole after 180 s, it is placed gently in the escape hole for 30 s. The escape is then covered. Subsequently, the rat is removed from the escape hole and returned to its home cage.
3. Before the next rat starts the trial, the platform surface is to be cleaned and disinfected using 10% (*w*/*v*) ethanol and then aired. Moreover, the platform is to be rotated randomly, but across all trials, the spatial position of the escape hole is kept constant for any given rat.
4. After a 10–15-min inter-trial interval, step 2 is to be repeated for any given rat.
5. Steps 1 through 5 are to be repeated with all rats for a 4-day period (in total, 10 trials per rat; 2 trials per day for 5 days).

#### Acquisition probe trial (day 6)

1. Twenty-four hours after the last training trial (day 6), the escape box is to be removed.
2. The rat is to be placed under the start box for 15 s, after which the start box is to be lifted and the buzzer is to be switched on.
3. The rat is to be permitted to explore the maze and holes for 90 s.
4. After the completion of the acquisition probe trial, the rats are to be allowed to rest in their home cages until the reversal learning trials are implemented.

#### Reversal learning trials (days 7–9)

1. Twenty-four hours after the completion of the acquisition probe trial, the reversal learning trials are to be started. Because the rats already know the extra-maze cues, as well as the surface of the platform, the habituation period need be omitted. For a given rat, the escape hole is to be rotated 180° from its original position and is to be kept constant throughout the entire reversal testing period.
2. Steps 1 through 5 outlined in “Acquisition phase (days 1–5)” are to be followed for three successive days (in total, 6 trials per rat; 2 trials per day for 3 days).

#### Reversal learning probe (day 10)

1. One day after the last reversal learning trial, steps 1 through 3 outlined in “Acquisition probe trial (day 6)” are to be followed.

## Concluding remarks

For assessing spatial learning and memory, BM has a wide range of applications. Among these are in experimental modelling of central nervous system disorders (e.g., genetic or pharmacologically induced models of Alzheimer disease), as well as in evaluating drugs with different pharmacological profiles or assessing the efficiency of specific diet regimens. Regardless of the protocol, whether used by others or self-modified, in any experiment utilizing the BM task, researchers should always follow approved protocols so as to gain as reliable data as possible, to allow experimental replication and enable communication. This approach will assure that spatial learning/spatial memory/cognitive flexibility impairments are genuine results that are replicable across laboratories.

In summary, when choosing a protocol and planning an experiment, (1) consider the specific aim of the experiment (e.g., spatial learning, working/reference memory, short/long-term memory, cognitive flexibility), (2) choose the appropriate test subjects (mice/rats, strain, genetic background), (3) make preliminary studies to standardize the protocol (e.g., aversive stimuli), (4) ensure that the conditions are maintained across trials and testing days, (5) bear in mind all the confounding factors (e.g., odor cues, decreased locomotor activity, anxiety) which may influence the performance of the test animals, and (6) once planned, obtain as much data as possible to fully and acutely characterize the performance of the test animals.

Taking into account that all the confounding factors which may influence the animal’s behavior in testing might be controlled and eventually excluded, we believe that, in regard to BM tasks, the advantages outweighs disadvantages.
